# Five-Years Angiographic Follow-Up of Wide-Neck Intracranial Aneurysms Treated With LEO Plus Stent

**DOI:** 10.3389/fneur.2021.744962

**Published:** 2021-11-26

**Authors:** José M. Pumar, Paula Sucasas, Antonio Mosqueira, Pedro Vega, Eduardo Murias

**Affiliations:** ^1^Catedra de Neurorradiología Intervencionista, Universidad de Santiago de Compostela, Santiago de Compostela, Spain; ^2^Neuroradiology Department, Hospital Clínico Universitario, Santiago de Compostela, Spain; ^3^Neuroradiology Department, Hospital Clínico Universitario de Oviedo, Oviedo, Spain

**Keywords:** aneurysm, stent, hemorrhage, embolization, LEO

## Abstract

**Background:** This study aimed to evaluate the angiographic and clinical outcome, with an emphasis on long-term follow-up, of the LEO Plus stent for wide-neck intracranial aneurysms treated in a single center.

**Methods:** We retrospectively examined a prospectively maintained database of patients treated with LEO Plus devices between January 2004 and December 2016. Data regarding patient demographics, aneurysm characteristics, and technical procedures were analyzed. Angiographic and clinical findings were recorded during the procedure and followed up over a period of at least 5 years.

**Results:** We identified 101 patients with 116 aneurysms. In 16 patients, the stent could not safely be placed. Thus, a total of 97 LEO Plus devices were implanted in 97 aneurysms of 85 patients. Adverse events (acute and delayed) were observed in 21.6% of cases (17/85), and most were resolved (70.6%; 12/17). Moreover, 5 years after the procedure, total morbidity and mortality were 2.3% (2/85) and 3.5% (3/85), respectively. Long-term imaging follow-up showed complete occlusions, neck remnants, and residual aneurysms in 73.1% (57/78), 14.1% (11/78), and 12.8% (10/78) of cases, respectively.

**Conclusions:** Long-term results of treatment of brain aneurysms with LEO stent show high rates of adequate and stable occlusion over time, with acceptable morbidity and mortality.

## Introduction

Endovascular management of wide-necked and/or complex intracranial aneurysms is therapeutically challenging and involves a high risk of recanalization, regrowth, and rerupture (1, 2). These lesions are difficult to treat, both surgically and endovascularly, because of their unfavorable geometry. Such a circumstance minimizes the possibility of achieving dense packing and elimination of aneurysms from circulation.

Currently, several stents are used for stent-assisted coil embolization. In this study, we presented our experience with stent-assisted coil embolization and stent-only therapy of wide-necked and/or complex intracranial aneurysms using the LEO Plus stent. We evaluated our results in terms of safety (i.e., technical success and morbidity and mortality rates associated with this device) and efficacy by assessing the impact of this technique on angiographic outcomes with an emphasis on long-term follow-up.

## Materials and Methods

### Patient Population and Aneurysm

This study was carried out as a retrospective review of our maintained database with regard to a consecutive series of patients with wide-necked intracranial aneurysms, treated with the LEO Plus stent between January 2004 and December 2016 at our institution. Patient selection for endovascular treatment was performed by a multidisciplinary team of interventional neuroradiologists, neurologists, and neurosurgeons. The treatment decision was based on the size, location, and morphology of the aneurysm, as well as the clinical status of the patient. Patients and their relatives were informed of the complications associated with the diagnosed condition, the treatment options available, and the risks. Written informed consent was obtained from every patient before treatment. The study was conducted with the approval of the Local Ethics Committee in compliance with national legislation and the Code of Ethics Principles for Medical Research Involving Human Subjects of the World Medical Association (Declaration of Helsinki).

The following data were collected: patient demographics and medical history, aneurysm characteristics, clinical presentation, procedural details and complications, and follow-up results of radiological data and functional outcomes. An aneurysm was considered wide neck if it was greater than 4 mm or if the dome-to-neck ratio was under 2. The degree of aneurysm occlusion was measured using the Raymond-Roy Grade Scale (1-complete occlusion, 2-neck remnant, and 3-residual sac) (3).

### Therapeutic Strategy

The patients received clopidogrel (75 mg/day) and aspirin (150 mg/day) for at least 7 days before the procedure. Tests for responsiveness to clopidogrel and aspirin were not available. In some cases, a loading dose of 300 to 600 mg clopidogrel and 300 mg oral aspirin or 0.5 to 1 g intravenous aspirin was used as an alternative. All procedures were performed after general anesthesia was induced and therapeutic heparinization was applied with activated clotting times of approximately 300 s. Dual antiplatelet medication, including clopidogrel (75 mg/day) and aspirin (150 mg/day), was continued for at least 6 months, followed by the long-term use of aspirin (150 mg/day).

### Endovascular Procedure

The LEO Plus stent deployment strategy is aimed at strict compliance with the recommendations of the LEO Plus device manufacturer (Balt Extrusion, Montmorency, France). The choice of LEO Plus stent was selected by three-dimensional angiography, stent length was determined as 30% longer than the aneurysm neck length, and the diameter was approximately the diameter of the parent artery. Deployment failure was assessed in terms of the following aspects: failure to advance through the delivery catheter, poor opening, poor positioning, and stent displacement.

Baseline and post-procedural imaging studies including digital subtraction angiography, MRI, or CT, performed after the procedure and at 1–3 months, 6 months, 1 year, 2 years, 3 years, and 5 years post-procedure, as reviewed by local investigators at our institution.

### Clinical Complications and Related Morbidity and Mortality

Periprocedural and postoperative complications were assessed. Clinical outcome was assessed at discharge and 1–3 months, 6 months, 1 year, 2 years, 3 years, and 5 years postoperatively using the modified Rankin scale (mRS); mRS scores ranging from 0 to 2 were considered reflective of a good clinical outcome. All complications were retrospectively revised by two senior investigators who determined the category of the event as “major” or “minor”. All major adverse events were considered when evaluating the overall incidence of neurological morbidity and mortality.

### Data Collection and Literature Review

Statistical analyses carried out included Student's *t*-test, Chi-square test, Mann-Whitney test, ANOVA, and multivariate analysis using IBM^®^ SPSS^®^ statistics version 22 (IBM Co., Armonk, NY, USA). For statistical analyses, values of *p* < 0.05 were considered statistically significant.

## Results

### Patients

Between January 2004 and December 2016, 101 patients with 116 aneurysms were selected for treatment with Leo Plus Stent. In 16 patients, the stent could not safely be placed. A total of 85 patients comprising 58 women (68.2%) and 27 men (31.8%) were treated endovascularly for 97 aneurysms by LEO Plus at our institution. The range, and mean, of age in this consecutive series of patients were 33 to 79, and 56.2 years, respectively.

A total of 57 patients (67%) were asymptomatic, whereas 28 (33%) were symptomatic; specifically, 21 patients (24.7%) underwent a diagnostic imaging test for non-specific symptoms such as headache or dizziness, 13 patients (15.3%) had cranial nerve deficit, and 6 patients (7.0%) had an ischemic stroke.

### Aneurysmal Characteristics

Most aneurysms (75.3%; 73/97) were located in the anterior territory while the rest (24.7%; 24/97) were located in the posterior territory. The most frequent location was the paraophtalmic segment of the internal carotid artery. In terms of aneurysm morphology, 85.7% were saccular, 11.2% were fusiform, and 3.1% were dissecting aneurysms. Regarding the size of the aneurysms, 6.41% were giant, 28.2% big, and 65.38% were small.

The mean aneurysm size was 8.5 mm (median, 8.6 mm; range, 2–42 mm). A total of 52 aneurysms (53.6%) had an unfavorable dome-to-neck ratio (<1.6), with a mean dome-to-neck ratio of 1.5.

### Intraprocedural Technique

Among 101 patients, stent deployment was successful in 96% (85/101) of cases. In 16 patients (15.8%), the safe deployment of the stent was not possible for various reasons, such as vascular tortuosity or difficult anatomy, acute vasospasm, and the instability of the embolization devices (Table 1).

**Table 1:** Summary of causes of unsuccessful deployment of stents and posterior treatment.

**N**	**Sex**	**Age (years)**	**Cause of no deployment of the stent**
1	♀	40	Vasospasm
2	♀	50	Unstable embolization devices
3	♂	52	Vascular tortuosity
4	♂	64	Vasospasm
5	♀	57	Vascular tortuosity
6	♀	44	Instability embolization devices vasospasm
7	♀	51	Vascular tortuosity
8	♂	53	Microwire placement was not achieved
9	♂	62	Difficult anatomy
10	♂	42	Vasospasm
11	♀	33	Vasospasm
12	♀	71	Instability of embolization devices
13	♂	33	Unstable embolization devices
14	♂	48	Vascular tortuosity
15	♀	59	Defect of stent deployment. Vascular tortuosity
16	♀	53	Incomplete aposition of the stent

A total of 73 devices were deployed in 73 patients with 73 aneurysms (85.9%). A total of 24 devices were used in 12 patients with a total of 24 aneurysms (14.1%) being treated.

### Clinical Outcome and Complications

The overall complication rate was 20% (17 patients). Periprocedural and subacute complications (until 7 days after the procedure) occurred in 13 patients (15.3%). Delayed complications occurred in four patients (4.7%). A total of 15 patients (17.6%) recovered ad integrum. Two patients (2.3%) had permanent deficits with important morbidity due to ischemic complications (mRS 3 and mRS 4, respectively), considered major adverse events, whereas the rest were considered minor events that resolved in less than 7 days

Most complications were due to thromboembolic events (14 patients; 16.5%), intracranial bleeding occurred in one patient (1.2%), one patient had a groin hematoma, and another had a groin hematoma plus a cervicofacial hematoma (2.3%) (Table 2).

**Table 2:** Complications of aneurysms treated with LEO Plus.

**No**.	**Sex**	**Age (years)**	**Complication**	**Timing**
1	♀	75	Basal ganglia ischemic event	4 days
			Cervical CIA pseudoaneurysm	2 years
2	♀	48	Stroke	2 days
3	♀	62	Right MCA stroke	30 minutes
4	♀	79	Left MCA stroke	2 days
5	♀	71	Acute thrombosis	Periprocedural
6	♀	53	Hemiparesia due to vasospasm	First 24 hours
7	♀	51	Vasospasm in cervical CIA	Periprocedural
8	♂	53	Vasospasm with lacunar ischemic lesions in CT	Periprocedural
			Groin hematoma	
9	♀	64	Vasospasm with intraarterial thrombus	Periprocedural
10	♀	49	Stent thrombosis in left MCA	12 days
11	♂	52	Stroke	4 months
12	♀	59	Stroke	1 day
13	♂	55	Thrombosis and migration of the stent	9 months
14	♀	69	Parietal intracranial parietal bleeding	Periprocedural
15	♀	41	Recent ischemic lesions with faciobrachial paresis 4 /5	Periprocedural
16	♀	62	Groin hematoma	6 days
			Cervico-facial hematoma due to lesion at external carotid artery	3 days
17	♂	42	Kinking of the stent	1 year

No significant difference was found between complications in the anterior territory (6/17; 35.3%) and the posterior (11/17; 64.7%). In relation to their size, complications were more frequent in the group of large aneurysms (8/17; 47.5%) and giant (3/17; 18.2%) than in small aneurysms (6/17; 35.3%). Complications were more frequent in saccular aneurysms (12/17; 70.5%) than in non-saccular ones (5/17; 29.5%).

The overall morbidity in our study, taking into account the patients with permanent deficits, was 2.3% (two patients), and the mortality rate was 3.5% (three patients). One death was due to early thrombotic stent occlusion immediately after the procedure, and the other patients died due to ischemic complications 2 days and 4 months after the procedure. In the present series, there were no patients who suffered from intracerebral bleeding.

### Occlusion Rate

Immediate post-procedural angiography indicated complete occlusion (Raymond-Roy class 1) in 39.8% of the aneurysms, class 2 occlusion in 15.9%, and class 3 occlusion in 44.3%.

The angiographic follow-up data were available for 78 patients with 78 aneurysms at 5 years. Complete occlusion was noted in 57 of 78 aneurysms (73.1%), residual aneurysm neck in 11 (14.1%), and recurrent aneurysms in 10 (12.8%).

With regard to the location of the residual aneurysms, the majority of the partial occlusions were noted in the lesions of the posterior territory; fewer residual aneurysms were noted in the anterior territory. In relation to the size of the residual aneurysms, most of the partial occlusions corresponded to large aneurysms and most were non-saccular. We found no relationship between the grade of occlusion and the size of the neck of the aneurysms, unlike most published series.

At follow-up, 11 patients showed recanalization, of which 6 of them required retreatment, while the rest were managed conservatively. In our study, most of the recanalizations occurred in the lesions of the anterior territory, probably because these were more frequent in our series (Table 3).

**Table 3:** Radiographic outcomes of aneurysms treated with LEO Plus.

**Follow-up timing**	**Grade of occlusion**	**%**
1–3 months	NFUA (*)	10,2% (9/97)
*N* = 88	1	39.8% (35/88)
	2	15.9% (14/88)
	3	44.3% (39/88)
6 months	NFUA (*)	15,5% (13/97)
*N =* 84	1	41.6% (35/84)
	2	17.9% (15/84)
	3	40.5% (34/84)
1 year	NFUA (*)	18,3% (15/97)
*N =* 82	1	51.2% (42/82)
	2	23.2% (19/82)
	3	25.6% (21/82)
2 years	NFUA (*)	18,3% (15/97)
*N =* 82	1	54.9% (45/82)
	2	23.2% (19/82)
	3	21.9% (18/82)
3 years	NFUA (*)	22,8% (18/97)
*N =* 79	1	55.7% (44/79)
	2	26.6% (21/79)
	3	17.7% (14/79)
5 years	NFUA (*)	24,3% (17/97)
*N =* 78	1	73.1% (57/78)
	2	14.1% (11/78)
	3	12.8% (10/78)

* *NFUA, No follow-up available*.

## Discussion

To date, mid- and long-term results after LEO Plus have been reported, demonstrating acceptable aneurysm occlusion and device navigability, as well as low associated morbidity and mortality rates. However, no long-term data has been reported in over 3 years. This study reported our experience with the LEO Plus stent, including the longest available follow-up in the literature. The results of the present series were further discussed in the context of the results reported in the literature regarding the outcome of treatment using intracranial stents (Table 4).

**Table 4:** Comparison of different studies (4–15).

**Author**	**Year**	**Single /Multicenter**	**Type of study**	**Number of patients**	**Number of aneurysms**	**Type of stent**	**Follow-up time**	**Total occlusion**
Kis et al. (10)	2005	Single center	Retrospective	21	25	Leo	3-12 months, average 5 months	56%
Lv et al. (6)	2010	Single center	Retrospective	28	28	Leo, Neuroform	3-24 months, mean 12 months	71.4%
Mc Laughlin et al. (7)	2013	Multicenter	Systematic review	656	702	Neuroform, Leo, Enterprise, Solitaire	Overall midterm 5.6-43.2 months (mean 6 months) 3 studies: greater than 24 months	71.9
Akmangit et al. (11)	2015	Single center	Retrospective	12	12	Leo baby, Enterprise	3 and 6 months	83.3
Machi et al. (12)	2015	Single center	Retrospective	29	29	Leo baby	MRI at 6 months Conventional DSA at 12 months	55%
Negrotto et al. (13)	2015	Single center	Retrospective	6	6	Leo baby, LVIS Jr	6 months	-
Lubicz et al. (4)	2017	Single center	Retrospective	50	52	Leo	Mean 50.2 months, range 12-139 months	76%
Lubicz et al. (8)	2017	Single center	Retrospective	49	53	Enterprise, Leo baby, LVIS Jr	3-84 months (mean 29 ± 19.6 months)	20.8%
Sedat et al. (9)	2017	Single center	Retrospective	153	155	Leo	8-36 months	85%
Voigt et al. (14)	2017	Single center	Retrospective	39	40	Leo	6 months	88.9%
Cagnazzo et al. (15)	2018	Single center	Retrospective	76	98	Leo	6-24 months, mean 14 months	70%
Djurdjevic et al. (5)	2019	Single center	Retrospective-Prospective	101	104	Leo baby	Mean 19.9 months	24.7%
Our study	2020	Single center	Retrospective-Prospective	85	97	Leo	At least 5 years >5 years in 20 patients	71.3%

### Technical Success

Treatment with the LEO Plus system presents specific problems, mainly related to difficulties in deployment. When LEO Plus devices were implanted in 85 patients, technical difficulties were encountered in 6.3% of cases, implying technical feasibility of 96%. Our failure rate is relatively low, similar to the 3.8% reported by Lubicz et al. (4), and is comparable to previously published series reporting stenting success rates ranging from 94 to 100% for the Enterprise stent, (16, 17) 93–100% for the Solitaire stent (18), and 89.4% (5) and 97.2% for the LEO stent (4). The study of Sedat et al. (9) reported a deployment failure of 1.9%. A direct comparison between our results and the results reported by these previous studies is not possible due to the variations in key factors such as patient selection criteria and population size.

### Clinical Complications and Related Morbidity and Mortality

The complication rates in our current study are consistent with those reported previously in similar studies, with total morbidity and mortality of 2.1 (2/85) and 3.5% (3/85), respectively. The rates of morbidity associated with the endovascular procedures of stenting and coiling greatly vary in the literature, ranging from 2.8 to 21% (19, 20). In our study, the rate of acute and subacute morbidity due to the procedure was 15.3%, delayed morbidity 2.1%, and permanent sequelae 2.1%. This contrasts with the absence of mortality and morbidity in a series of 50 patients receiving a LEO stent reported by Lubicz et al. (4) and is slightly higher than the 10% observed by Lv et al. (6) following treatment of 28 patients with a LEO stent (6) and the series of Sedat et al. (9) of 153 patients who reported a morbidity rate 30 days after the procedure of 9.15%. Regarding mortality, we found a procedural-related mortality rate of 3.1%, in contrast to the 1.8% reported by McLaughlin et al. (7) and the non-observation of mortality reported in the patients studied by Lubicz et al. (4, 8) Sedat et al. (9) Kis et al. (10) and Djurdjevic et al. (5).

Thromboembolic events were the most frequent complications of endovascular treatment despite the dual antiplatelet treatment routinely administered by nearly all neurointerventional teams. Among these complications, the most serious is usually stent thrombosis. The study of Lv et al. (6) observed three (10%) acute occlusions of the parental artery after deployment in 28 LEO-treated patients, suggesting a thrombogenic characteristic of the stent. Meanwhile, Djurdjevic et al. (5) reported one patient with an acute in-stent thrombosis with important clinical impact (mRS 4) and a delayed in-stent thrombosis in three patients with severe morbidity (mRS 5) in one of those patients. Although stent thrombosis was observed less frequently in our study acute (2%; 2 patients of 97 treated aneurysms), we agree with the recommendation by Lv et al. (6) that the LEO stent should never be used without antiplatelet treatment and should not be used for the treatment of ruptured aneurysms as the first choice.

### Rate Occlusion

In our study, initial angiographic results (1–3 months) showed complete occlusion (Raymond 1) in 39.8% of the aneurysms, which was similar to the complete occlusion rate of 41% reported by Murayama et al. (22). This is also consistent with previous reports, (4, 7, 9, 23, 24) and slightly better than other case series (8).

The angiographic follow-up final, at 5 years, demonstrated complete occlusion in 57 aneurysms (73.1%), showing occlusion rates increasing from 43.8 to 73.1%. This progressive occlusion has been described in several studies, (25) with a frequency of 14 to 57%, (26, 27) and similar results were observed in our study after a LEO stent was used. Similar to Sedat et al. (9) we think that progressive occlusions may be due to hemodynamic changes and the probable induction of endothelization, produced by the flow diversion effect of LEO Plus, which could lead to long-term stable occlusion.

In our study, we found a recanalization rate of 11.3% of treated aneurysms, slightly lower than the 20 and 17.5% reported by Lubciz et al. (4) and Maldonado et al. (21) respectively, and similar to that reported by Geyik et al. (10%) (28) and Sedat et al. (8.4%) (9). The retreatment rate in our study was 6.2%; these results were slightly higher than the previously published rates of 4 and 5.7% by Sedat et al. (9) and Djurdjevic et al. (5) respectively and similar to other reported series (2, 10, 29, 30). We believe these differences may be because our study implemented one of the longest follow-ups (5 years) to date.

This study has several limitations, the most significant of which is the fact that it was carried out as a single-center non-randomized retrospective series. The results reflect a single-operator and single-center experience, and results may not be generalizable to other centers. However, the data do reflect consecutive experiences from the use of the LEO Plus device with very long-term follow-up, providing important data on clinical and anatomical stability.

In conclusion, long-term results of treatment of brain aneurysms with Leo stent show high rates of adequate and stable occlusion over time, with acceptable morbidity and mortality.

## Data Availability Statement

The original contributions presented in the study are included in the article/supplementary material, further inquiries can be directed to the corresponding author/s.

## Ethics Statement

The studies involving human participants were reviewed and approved by Comite Etico SERGAS. The patients/participants provided their written informed consent to participate in this study.

## Author Contributions

JP, AM, and PV: conception and design of the study. AM and PS: data acquisition and analysis. JP: manuscript drafting. PV and EM: critical revision. All authors have reviewed and approved the manuscript.

## Conflict of Interest

The authors declare that the research was conducted in the absence of any commercial or financial relationships that could be construed as a potential conflict of interest.

## Publisher's Note

All claims expressed in this article are solely those of the authors and do not necessarily represent those of their affiliated organizations, or those of the publisher, the editors and the reviewers. Any product that may be evaluated in this article, or claim that may be made by its manufacturer, is not guaranteed or endorsed by the publisher.
